# Crosstalk Between Cancer Associated Fibroblasts and Cancer Cells in Scirrhous Type Gastric Cancer

**DOI:** 10.3389/fonc.2020.568557

**Published:** 2020-10-16

**Authors:** Yuichiro Miki, Masakazu Yashiro, Lidia Moyano-Galceran, Atsushi Sugimoto, Masaichi Ohira, Kaisa Lehti

**Affiliations:** ^1^ Department of Gastroenterological Surgery, Osaka City University Graduate School of Medicine, Osaka, Japan; ^2^ Department of Microbiology, Tumor and Cell Biology, Karolinska Institutet, Stockholm, Sweden; ^3^ Department of Biomedical Laboratory Science, Norwegian University of Science and Technology, Trondheim, Norway

**Keywords:** cancer associated fibroblast, gastric cancer, tumor microenvironment, scirrhous carcinoma of the stomach, fibroblast growth factor receptor, transforming growth factor β1

## Abstract

Gastric cancer (GC) is the third leading cause among all cancer deaths globally. Although the treatment outcome of GC has improved, the survival of patients with GC at stages III and IV remains unsatisfactory. Among several types of GC, scirrhous type GC (SGC) shows highly aggressive growth and invasive activity, leading to frequent peritoneal metastasis. SGC is well known to accompany abundant stromal cells that compose the tumor microenvironment (TME) along with the produced extracellular matrix (ECM) and secreted factors. One of the main stromal components is cancer associated fibroblast (CAF). In the SGC microenvironment, CAFs are a source of various secreted factors, including fibroblast growth factors (FGFs), which mediate prominent tumor-stimulating activity. In turn, cancer cells also secrete numerous factors, which can activate and educate CAFs. Current findings suggest that cancer cells and stromal cells communicate interactively *via* the soluble factors, the ECM, and likely also by exosomes. In this review, we focus on the soluble factors mediating communication between cancer cells and CAFs in SGC, and consider how they are related to the modulation of TME and the high rate of peritoneal metastasis. At last, we discuss the perspectives on targeting these communication pathways for improved future treatment.

## Introduction of Scirrhous GC Microenvironment

Gastric cancer (GC) is diagnosed with 5^th^ frequency among all cancers and is the third-leading cause of cancer death, with one million new cases and nearly 800,000 deaths globally in 2018 (1). Although the survival outcome has been improved by early screening, optimal surgery, chemotherapy, and molecular targeted therapy, the median overall survival is reported to be only 10–16 months in patients with metastatic or unresectable GC (2–5).

**Figure 1:**
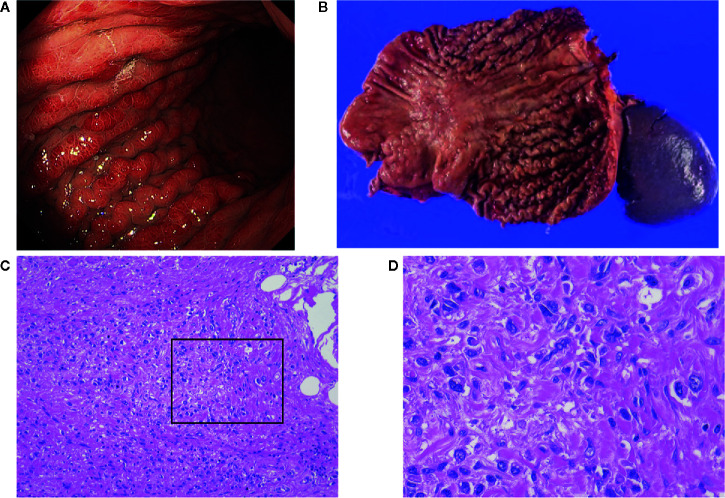
**(A)** Endoscopic view of scirrhous type gastric cancer (SGC); **(B)** Image of resected specimens of total gastrectomy. Tumors did not have marked ulceration or raised margins, the gastric wall was thickened, showing typical macroscopic view of SGC. **(C, D)** Microscopic images from the specimen shown in **(B)**. Cancer cells are invading into the stroma containing fibroblasts and extracellular matrix.

Scirrhous gastric cancer (SGC), an aggressive subtype of GC, shows rapid infiltration in the gastric wall, progressive invasion into the serosal layer, and seeding to the peritoneum. GC classification into 6 types (type 0–5) by macroscopic features, as Borrmann proposed (**Table 1**), has been used clinically. On the other hand, Laurén classified GC into two main subtypes of intestinal type (differentiated type) and diffuse type (undifferentiated type) by microscopic features (6). By using these two classifications, SGC can be defined as macroscopic Borrmann type 4 and microscopic diffuse type (**Figure 1**). The incidence of SGC was 7.5% (284/3,842) in the registry of our institute (unpublished data), which is consistent with the Japanese nation-wide registry data (6.6%) (7). The Cancer Genome Atlas (TCGA) Research Network has suggested a molecular-based classification of GC into four subtypes: 1) the Ebstein–Barr Virus positive tumors (EBV 8%), with frequent PI3KCA mutations, high DNA hypermethylation, *JAK2/PDL1* amplification and *PDL2/CDKN2A* silencing; 2) MicroSatellite Unstable tumors (MSI 22%), with high rates of mutations, including genes that encode oncogenic proteins; 3) genomically stable tumors (GS 20%), characterized by diffuse histology, mutations in CDH1/RHOA and fusions in the CLDN18 family; and 4) tumors which have chromosomal instability (CIN 50%), characterized by intestinal histology and amplification of several tyrosine-kinase receptor genes (8). Most SGCs can be classified into the GS group defined by the TCGA classification, although there is insufficient data regarding this issue (8).

**Table 1:** Macroscopic type of gastric cancer according to Japanese Gastric Cancer Association criteria.

Macroscopic type	Description
Type 0 (superficial)	Typical of T1 tumors.
Type 1 (mass)	Polypoid tumors, sharply demarcated from the surrounding mucosa.
Type 2 (ulcerative)	Ulcerated tumors with raised margins surrounded by a thickened gastric wall with clear margins.
Type 3 (infiltrate ulcerative)	Ulcerated tumors with raised margins surrounded by a thickened gastric wall without clear margins.
Type 4 (diffuse infiltrate)	Tumors without marked ulceration or raised margins, the gastric wall is thickened and indurated and the margin is unclear.
Type 5 (unclassifiable)	Tumors that cannot be classified into any of the above types.

The prognosis of SGC is worse than it is for the other types, and the 5-year survival rate of Japanese patients with macroscopic type 4 is reported to be 17.7%, while for all registered patients the Japanese Gastric Cancer Association (JGCA) registry reports a 5-year survival of 68.9% (9). An Italian group reported worse survival data for this sub-population of GC, and 5-year survival rate after R0 resection was reported to be only 4% (10). These unsatisfactory survival outcomes are partly because of diagnostic difficulties in early stage due to rapid growth, which leads to low percentage of curative resection for patients with SGC. Even when we perform standard treatment with curative intent, SGC often recur with peritoneal metastasis, which frequently develop resistance to chemotherapy as well as available molecular targeted therapy. Possibly, small cytological lesions in the peritoneum grow and generate a fibrotic microenvironment that may later interfere with drug delivery to the cancer cells.

SGC cell proliferation is coupled with remarkable fibrosis when the cancer cells enter into the submucosa composed of stromal cells. Coincidentally, the fibrosis is induced by the excessive deposition of collagen (COL), including COL1A1, COL1A2, COL3A1, COL4A1, COL4A2, COL5A1, and COL5A2 in GC (11). The unique feature of SGC, compared to the other GC types, is high expression of type IV collagen in the stroma of undifferentiated GC with desmoplastic reaction (40.4% vs 9.0%) (12). The distinctive histological findings of rapid tumor enlargement with fibrosis imply that the growth of the fibrotic tumor microenvironment (TME) can be controlled by intercellular communication between the SGC cells and the stromal cells. One of the main cellular components of SGC microenvironment is cancer associated fibroblasts (CAF). CAFs secrete various molecules, such as fibroblast growth factor (FGF), which directly stimulate cancer cells. Conversely, cancer cells also secrete factors which can activate and educate CAFs. While it has been well established that cancer cells and CAFs communicate interactively through soluble factors, the communication *via* exosomes has only been recognized more recently (13, 14). In this review, we will focus on the reciprocal communication between cancer cells and CAFs in SGC, as well as the relevance of this communication to both the remodeling of TME and the high rate of peritoneal metastasis. Finally, we will discuss the perspectives on future treatment targeting these communication pathways/mechanisms.

## TME COMMUNICATIONS *via* Soluble Factors

### FGF-FGFR Axis

Fibroblast growth factors (FGFs) regulate various cellular processes, such as stemness, proliferation, apoptosis evasion, migration, and invasion (see **Table 2**) (15, 21, 30–32).

**Table 2:** FGFs classification according to their functions in cancer progression.

Function	FGFs (reference)
Proliferation	FGF1 (15), FGF2 (16), FGF3 (15), FGF7 (17, 18), FGF10 (19)
Stemness	FGF2 (20)
Apoptosis evasion	FGF2 (21), FGF9 (22)
Migration and invasion	FGF2 (16)
Angiogenesis	FGF1 (15, 23), FGF2 (24), FGF3 (15), FGF4 (25), FGF8 (26), FGF18 (27)
Resistance to therapy	FGF1 (28), FGF2 (28), FGF3 (29), FGF4 (29), FGF19 (29)

The FGF family includes 22 secreted factors, which are divided into seven subgroups according to their phylogenetic relation, homology, and biochemical function (33). Members of five FGF subfamilies: FGF1 (FGF1, FGF2), FGF4 (FGF4, FGF5, FGF6), FGF7 (FGF3, FGF7, FGF10, FGF22), FGF8 (FGF8, FGF17, FGF18), and FGF9 (FGF9, FGF16, FGF20) are released to function in paracrine and autocrine manner. On the other hand, the FGF15 (FGF15, FGF19, FGF21, FGF23) subfamily is produced by endocrine glands as secreted hormones for metabolic modulation with α- and β-Klotho family proteins. In contrast, FGF11, FGF12, FGF13 and FGF14 lack secretory N-terminal peptides that direct newly produced proteins to secretory pathway, and thus remain intracellular (33).

As for the receptors of the FGF ligands, four distinct FGF receptors (FGFRs), FGFR1 (Flg), FGFR2 (K-sam), FGFR3, and FGFR4 exist, and if deregulated can function as oncogenes to drive specific cancer types including GC (17, 34–36). For example, we have previously shown that FGFR4 interaction with the membrane-type matrix metalloproteinase MT1-MMP increases both FGFR4-FRS2-Src kinase signaling and MT1-MMP-driven cancer cell invasion in a Gly388Arg SNP dependent manner (36). Canonically, FGFRs are monomers in their inactive state, and the binding of FGF ligands triggers receptor dimerization. The binding of FGF to FGFR causes activation of the receptor *via* cross-phosphorylation of the intracellular kinase domains. This leads to the recruitment of adaptor and scaffold proteins, and biochemical signals are transduced by activated FGFRs into cytosolic signaling cascades. Among four FGFRs, FGFR2 is identical to the K-*sam-II* gene, and it was originally identified in an extract from the SGC cell line KATO-III (37). We can observe this amplification of *FGFR2* in OCUM-2M, which was also established from patients with SGC (38).

Gastric cancer with *FGFR2* amplification is significantly associated with poor survival outcome. Although *FGFR2* amplification has been found in 5–10% of GC, the ratio is significantly higher in diffuse type (including SGC) (8), suggesting that *FGFR2* amplification is one of key factors in the most aggressive SGC.

FGFR2 isoforms IIIb and IIIc are mainly expressed in epithelial and mesenchymal tissues (39–41). In general, the FGFR2 IIIb isoform binds FGF3, FGF7, and FGF10 with high affinity, while the IIIc isoform has preference for FGF2, FGF4, and FGF20 (42, 43). It has also been reported that FGF10 and FGFR2-IIIb promote proliferation and patterning of the forestomach, and are involved in early epithelial growth before differentiation (44).

Despite these general findings, there are only a few studies regarding FGF-FGFR axis particularly in SGC. Yashiro et al. identified that the growth-stimulating factor from gastric fibroblasts to SGC cells is FGF7 (17). FGF7 stimulates the growth of SGC cells, but not that of well-differentiated adenocarcinoma cells. Since *FGFR2* amplification is more often observed in SGC than non-SGC, FGF-7 secreted by gastric fibroblasts is significant in the progression of SGC with *FGFR2* amplification in a paracrine manner. This was supported by the report from Huang et al., which described that FGF7/FGFR2 increase invasion and migration of GC cells through a thrombospondin 1 (THBS1)-mediated pathway. Increased expression of THBS1, an extracellular glycoprotein that has multiple roles in cell-matrix and intercellular interactions (45), significantly correlated with tumor differentiation.

Sun et al. reported that CAF-secreted and MMP7-activated FGF9 promotes apoptosis evasion and invasive ability of gastric cancer cells (22). MMP7 not only has the potential to degrade the extracellular matrix, but also promotes apoptosis evasion in cancer cells. In a Chinese GC cohort study, FGF9 was also associated with accelerated proliferation and apoptosis inhibition of GC cells in an autocrine manner (46).

### TGFBR Axis

**Figure 2:**
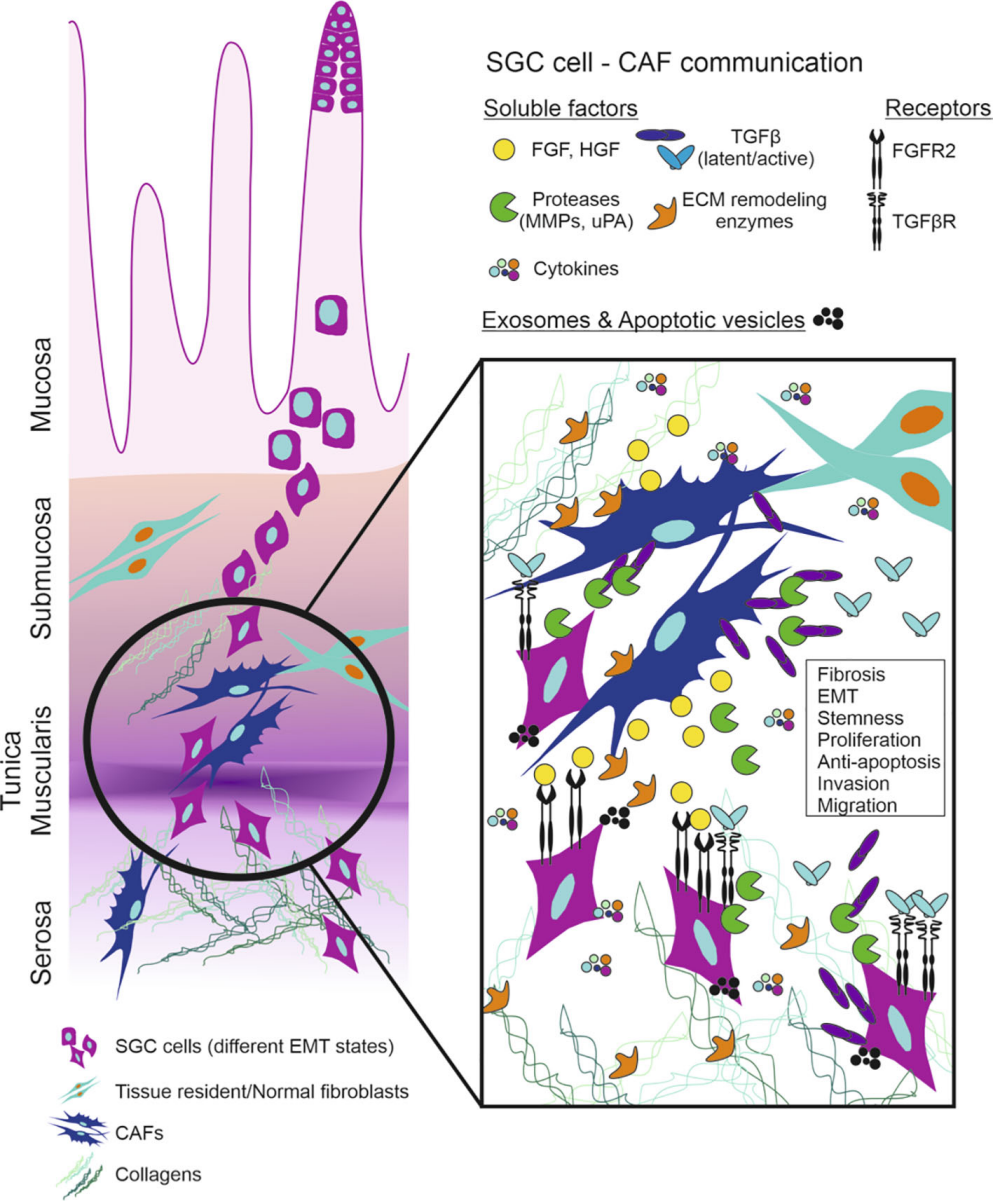
Schematic representation of SGC cells invasion and communication with CAFs. FGF-FGFR and TGFβ-TGFβR axis are the main players in the tumor microenvironment of SGC. SGC (scirrhous gastric cancer), CAF (cancer associated fibroblasts), FGF (fibroblast growth factor), FGFR (FGF receptor), HGF (hepatocyte growth factor), MMP (matrix metalloproteinases), uPA (urokinase-type plasminogen activator), TGFβ (transforming growth factor β), TGFβR (TGFβ receptor), ECM (extracellular matrix), EMT (epithelial-to-mesenchymal transition).

Transforming growth factor β (TGFβ) produced by fibroblasts increases the invasive capabilities of SGC cells (47) (**Figure 2**). Whole exome and RNA sequencing analyses comparing CAFs and normal fibroblasts (NF) revealed that many of the genes with upregulated expression in CAFs were associated with TGFβ1 (TGFB1) pathway (48).

In the conditioned medium from fibroblasts, TGFβ is mainly in a latent form, whereas its active form is detected in the conditioned medium from GC cells (47, 49). Proteases such as plasmin and cathepsin can activate the latent TGFβ (50). Most GC cells secrete urokinase-type plasminogen activator (uPA) which converts latent TGFβ to active TGFβ (51, 52). Our group previously reported that SGC cells derived from peritoneal metastasis (OCUM-2D) produced six times higher amounts of uPA than SGC cell line (OCUM-2M) which was established from primary lesion of the same patient (53), suggesting the possible role of uPA in peritoneal metastasis of SGC. The latent TGFβ from gastric fibroblasts and SGC cells is activated by uPA from SGC cells. It has been shown that TGFβ promotes collagen synthesis not only by fibroblasts but also by cancer cells, resulting in diffuse fibrosis of SGC (54).

Although the effect of TGFβ on tumor growth is controversial, Komuro et al. used a SGC cell line (OCUM-2MLN) and showed that disruption of TGFβ signaling in SGC may accelerate tumor growth through upregulated tumor angiogenesis that is induced by decreased expression of THBS1 after inhibition of TGFβ signaling by dominant-negative TGFBR (55).

TGFβ produced by either gastric fibroblasts or cancer cells affects the invasive capabilities of SGC cells by inducing a morphologic change of the cells to a spindle shape, a process known as the epithelial-to-mesenchymal transition (EMT) (56). Cancer cells experiencing EMT develop invasive and migratory capabilities (57–59). Shinto et al. revealed that TGFβ significantly upregulates the activity of RhoA and myosin light chain-2 phosphorylation, whereas TGFβ1 decreases ZO-2 and E-cadherin in SGC cells. Moreover, the TGFBR kinase inhibitor Ki26894 inhibited both invasion and EMT in SGC cells. It has been also revealed that the combination of S-1 (Tegefur/Gimeracil/Osteracil, 5-FU derivative) and Ki26894 decreases tumor growth and lymph node metastasis more effectively than Ki26894 alone. We have confirmed that TGFβ produced by CAFs increased the migration and invasion ability of cancer cells derived from SGC (60). Our group has also shown that hypoxia stimulates EMT in SGC cells *via* autocrine TGFβ signaling (61).

Ishimoto et al. reported that CAFs express high levels of Rhomboid 5 homolog 2 (RHBDF2). Expression of RHBDF2 in fibroblasts is prompted by inflammatory cytokines secreted by SGC cells. RHBDF2 promotes cleavage of TGFBR by activating a disintegrin and metallopeptidase domain 17 (ADAM17, also called TACE) and motility of CAFs in response to TGFβ1. They reported that these CAFs with high motility can also increase the invasion of SGC cells into extracellular matrix and lymphatic vessels in nude mice (48). Kawajiri et al. revealed that A-77, another TGFβ inhibitor, decreased the invasion capability of SGC by decreasing the intercellular interaction between SGC cells and CAFs, in conjunction with decreased tumor growth and dissemination in an intraperitoneal tumor model.

In addition to the function of TGFβ in the regulation of invasiveness, CAFs regulate cancer cell stemness in SGC. Conditioned medium (CM) from CAFs can significantly increase spheroid colonies and the expression of cancer stemness markers of SGC cells. These stimulating activities by CM are significantly abolished by TGFβ inhibitors, but not by FGFR and c-Met inhibitors. Thus, TGFβ from CAFs is considered to be an important factor to sustain stemness in SGC (62).

On the other hand, TGFβ from SGC surrounding CAFs increases α-smooth muscle actin (a-SMA) expression in fibroblasts thorough SMAD pathway, suggesting that SGC cells can reprogram NFs into CAFs (63). This means that SGC cells can educate CAFs to sustain the favorable microenvironment, and TGFβ plays a critical role in this inter-cellular communication.

### HGF, Matrix Metalloproteases, and Cytokines

CAFs also produce HGF, which is a known regulator of the SGC cell invasiveness. The *c-met* gene encoding the HGF receptor c-Met is amplified more frequently in SGC than in non-scirrhous gastric cancer (64). HGF is not usually detected in the CM from gastric cancer cells, thus, it can affect the invasive capabilities of SGC cells in a paracrine fashion (**Figure 2**). In diffuse-type gastric carcinoma, E-cadherin is frequently down-regulated by methylation and mutations, a phenomenon that may be related to tumor invasion (65). Additionally, another adherens junction protein, Desmoglein-2 is down-regulated in diffuse-type gastric cancer (66, 67). Sank-Uk Han reported that exposure of SNU-16 gastric cancer cells to HGF down-regulates the expression of E-cadherin, and induces morphological changes from epithelial to mesenchymal type (68). Thus, HGF could be one of the main factors which control cell-cell adhesion in SGC.

Tendo et al. reported that COX2 inhibitor in combination with S-1 (5-FU derivative) decreases the production of HGF in CAF, and suppresses tumor growth and lymph node metastasis in SGC mouse model. It has also been reported that the HGF antagonist NK4 can effectively inhibit the progression of peritoneal metastasis of SGC, revealing c-Met as a promising target candidate to halt SGC progression.

In addition to secreting growth factors, fibroblasts produce matrix metalloproteinases (MMPs) that allow cancer cells cross tissue boundaries (69, 70). In early stages, cancer cells growing at the mucosa need to invade into the submucosa beyond the muscularis mucosae. Extracellular matrix degradation and loss of cell-cell adhesion facilitate the tumor invasion. MT1-MMP on the surface of GC cells activates MMP-2 produced by fibroblasts (71). Therefore, MMP2 from the stromal cells may affect cancer progression in a paracrine manner, even though at the early stage cancer cells are separated from stromal cells by the basement membrane.

Lysyl oxidase (LOX) is produced as a 50-kDa proenzyme (pro-LOX). This pro-LOX is secreted and then cleaved by bone morphogenetic protein 1 in the extracellular space to form a 30-kDa mature enzyme and an 18-kDa pro-peptide (LOX-PP) (72, 73). LOX and LOX-like 1-4 oxidize lysine residues in collagens and elastin (74), leading to covalent cross-linking and stabilization of these ECM structural components, conferring much of the tensile strength to collagen and elastic fibers (75). Kasashima et al. reported that the expression of LOX in GC cells affects the EMT in hypoxic conditions (76). Furthermore, CAFs produce more LOXL2 than normal gastric fibroblasts, which increases the invasive capability of SGC cells in a paracrine manner (77).

Since the origin of CAFs of SGC remained uninvestigated, our group tackled the issue. Conditioned medium from SGC cells significantly increased twofold or threefold the migratory ability of bone marrow mesenchymal cells (BM-MCs) but not of non-SGC cells. This implied that BM-MCs were preferentially recruited by a factor(s) from SGC cells. To confirm the molecules increasing the homing capability of BM-MCs, chemokines were screened using a protein array and the protein production level between diffuse type GC cells and non-diffuse type GC cells were compared. Seven out of 102 screened chemokines (CXCL1, CXCL5, lipocalin-2, CXCL8, Dkk1, CCL20, and EMMPRIN) were expressed in SGC but not in the non-SGC cell lines MKN74 and SNU16. Among them, only CXCL1 significantly increased both the invasion capacity and motility of BM-MCs. Thus, we concluded that BM-MCs are recruited to SGC TME *via* CXCL1-CXCR2 signaling (78).

## Extracellular Vesicles, miRNA

Exosomes are small vesicles with a diameter of ~30–100 nm originated from the endosomal system during formation of multivesicular bodies (79). In cancer, exosomes have been implicated in proliferation, angiogenesis, immunosuppression, and preparation of premetastatic niches in secondary organs (80). Various studies have reported that exosomes mediate local and systemic cellular communication through the transfer of information *via* microRNAs, long non-coding RNAs (lncRNAs), mRNAs, proteins, metabolites and other substances.

Although their model was not gastric cancer, Webber et al. reported that exosomal TGFβ can differentiate fibroblasts into CAFs. At the surface of exosomes, TGFβ elicits SMAD-dependent signalling (13). Thus, exosomal TGFβ may be related to the differentiation of CAFs in SGC.

Naito et al. investigated the miR-143 expression in SGC and non-SGC, and reported that miR-143 expression is significantly higher in SGC tissue than in non-SGC tissue. They also showed that miR-143 enhances the expression of collagen type III in normal gastric fibroblasts and CAFs by activation of TGFβ/SMAD signaling, suggesting that miR-143 and TGFβ signaling regulate fibrosis of SGC tissue.

Our group showed that CD9 expression is higher in CAF-secreted exosomes than in NFs exosomes, and that CAF-secreted exosomes are taken up by SGC cells, but not by the other types of GC cells. Exosomes from CAFs stimulate the migration and invasion of SGC cells, which is inhibited by antibody or siRNA against the exosomal CD9. Interestingly, MMP2 expression in SGC cells is decreased by CD9-siRNA. Thus, we concluded that CD9-positive exosomes from CAFs stimulate the MMP2 expression and migration ability of SGC cells (81).

The role of miRNA in GC has been reported in the context of FGF-FGFR signaling. FGF18 is overexpressed in genomically stable and chromosomal instable GC subtypes, where it is associated to poor patient survival. Similarly, FGF18 is upregulated in seven out of eleven (63.6%) GC cell lines. Knocking down FGF18 inhibits tumor formation capabilities, induces cell cycle arrest in G1 phase and enhances drug sensitivity. In this report, miR-590-5p was identified as a direct target of FGF18, implying that FGF18 secretion can be regulated by exosomal miRNA (82).

Exosomal miRNA from CAFs may be related to chemo-resistance of GC cells. It was recently reported that exosomal miR-522 secreted by CAFs targets ALOX15 and blocks lipid-ROS accumulation in cancer cells, inhibiting ferroptosis and resulting in decreased chemo-sensitivity (83).

Finally, we will refer to the function of apoptotic vesicles from cancer cells. Cancer cells co-invade with CAFs, and CAF invasion often precedes invasion by cancer cells, resulting in CAF-led cancer cell invasion. When cancer cells interact with CAFs by death receptor 4, caspase-8 is activated in cancer cells and leads to apoptosis. Apoptotic cancer cells conversely release apoptotic vesicles and stimulate invasion of CAFs. In CAF-led cancer invasion, cancer cells move through tunnels in the substrate made by the leading CAFs. It has also been reported that cancer cells become highly motile along collagen bundles and these bundles are used as ‘highways’ for efficient migration. This may be one of the mechanisms responsible for the highly invasive characteristics of SGC (84).

## High Rate of Peritoneal Metastasis; Relation to EMT, Niche Formation

The most frequent type of metastasis in GC is peritoneal metastasis. The peritoneum constitutes a superficial monolayer of mesothelial cells and submesothelial stromal tissue, in which cancer-stroma interactions occur (85). Fibroblasts at peritoneal metastatic sites contribute to tumor progression.

**Figure 3:**
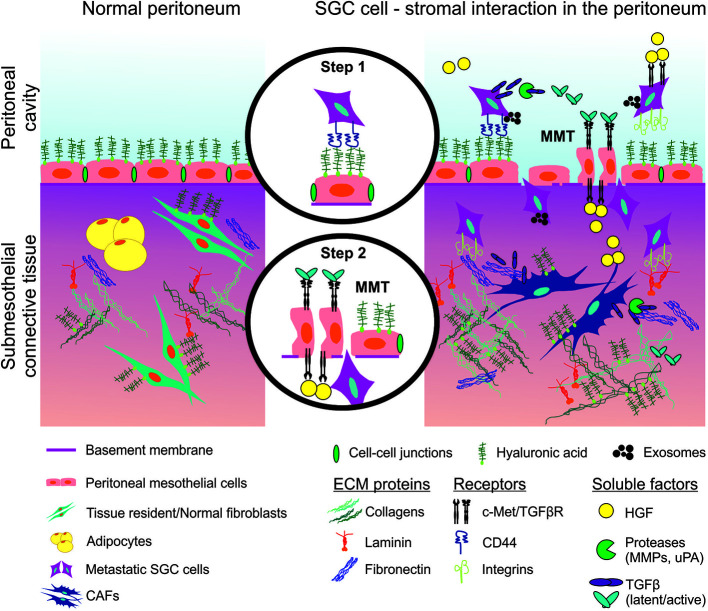
Schematic representation of peritoneal invasion by SGC cells and communication with the stroma. Step 1: interaction between SGC cells and peritoneal mesothelial cells (PMCs). Step 2: PMCs undergo mesothelial-to-mesenchymal transition (MMT) and allow SGC cells to invade the submesothelial connective tissue. SGC (scirrhous gastric cancer), CAF (cancer associated fibroblasts), ECM (extracellular matrix), TGFβ (transforming growth factor β), TGFβR (TGFβ receptor), HGF (hepatocyte growth factor), c-Met (HGF receptor), MMP (matrix metalloproteinases), uPA (urokinase-type plasminogen activator).

The initial step of peritoneal metastasis is the adhesion of cancer cells to the peritoneal mesothelial cells (PMCs), followed by the exfoliation of these mesothelial cells and the adhesion to the submesothelial connective tissue (**Figure 3**). The interaction between cancer cells and PMCs is mediated by the adhesion molecule CD44 expressed on the cancer cells and the hyaluronic acid expressed on PMCs surface (86). Stromal fibroblasts increase CD44 expression of GC cells through TGFβ signaling, thus stimulating the adhesion of SGC cells to the mesothelium (87). Another important step in peritoneal dissemination is the adhesion of cancer cells to the submesothelial components. The main components of the submesothelial matrix are laminin, fibronectin, type IV collagen at the basement membrane and type I collagen in the underlying interstitial matrix. Cancer cells adhere to these components *via* integrins, in particular α2β1- and α3β1-integrins (88). In the peritoneal cavity, GC cells which disseminated from the primary tumor are usually exposed to low oxygen levels (89). Experimentally, hypoxic (1% O_2_) conditions increase the adhesion capability of SGC cells, compared to normoxic (21% O_2_) conditions. Under hypoxia, TGFβ increases the expression of α2-, α3-, and α5-integrin in GC cells, promoting adhesion to the peritoneum. This can partially explain the high metastatic potential of SGC cells.

Upon peritoneal metastasis, a monolayer of PMCs that lines the peritoneal cavity undergoes mesothelial-to-mesenchymal transition (MMT). TGFβ from GC cells promotes morphological changes in mesothelial cells and thus likely associates with peritoneal dissemination (90). In fact, when mesothelial cells are exposed to fibroblasts, they become hemispherical and separated from each other, while unexposed mesothelium remains a flat monolayer. Both cancer cells and host fibroblasts stimulate morphological changes in mesothelial cells (91). HGF produced by peritoneal fibroblasts is associated with the morphology of mesothelial cells in monolayers so that the resulting microenvironment becomes suitable for the peritoneal dissemination of cancer cells (92).

Regarding exosomal communication, an investigation of exosomal miRNA profiles in peritoneal fluid showed that miR-21-5p was highly expressed in GC with serosal invasion. These findings suggest that miR-21-5p may be one candidate biomarker of peritoneal metastasis after GC resection. Exosomal miR-21-5p derived from GC cells was proven to induce MMT by activating TGFβ/SMAD pathway by alleviating the inhibitory action of SMAD7 (93).

## Perspective on Treatment

### FGFR2

Since previous data suggests that up-regulation of FGFR2 signaling is critical in a subset of GC patients including SGC, precision medicine approaches targeting FGFR2 by specifically designed drugs have recently emerged. Anti-FGFR2 specific monoclonal antibodies, FGF traps, and selective and non-selective FGFR inhibitors are among these drugs.

### Monoclonal Antibodies, FGF Traps

There are several antibodies which have shown promise in pre-clinical studies (**Table 3**). Among trials with them, bemarituzumab (FPA144) have provided preliminary data suggesting promising efficacy in patients with GC.

**Table 3:** Drugs targeting FGFRs.

Drug	Mechanism	Clinical data	Ongoing trial
Bemarituzumab (FPA144)	FGFR2 IIIb antibody	5 PR out of 28 patients with high FGFR2b overexpressing GEA	Phase III trial (FIGHT) (NCT03343301)
FP-1039 (GSK3052230)	FGF trap	No objective responses in phase I study for patients with advanced solid tumor	
NSC12	FGF trap	Only preclinical data	
AZD4547	FGFR1/2/3 inhibitor	*FGFR2* amplification cohort: 1PR, 4SD	SHINE trial (NCT01457846)
BGJ398	FGFR1/2/3 inhibitor	No data for GC	Phase I
E7090	FGFR1/2/3 inhibitor	Only one GC patient enrolled in the study showed PR	Phase I/II (NCT02275910)
LY2874455	Pan-FGFR inhibitor	1 PR, 12SD/29 patients	
JNI-42756493(Erdafitinib)	Pan-FGFR inhibitor	Safety profile was shown in phase I	Phase II
Lucitanib	Multi kinase inhibitor	7 responses/27 patients	Phase II
ARQ087 (Derazantinib)	Multi kinase inhibitor	3PR/18 evaluable patients with FGFR genetic alterations	
Dovitinib	Multi kinase inhibitor	1 PR, 2SD/14 evaluable patients	(NCT01719549)

FGFR, fibroblast growth factor receptor; FGF, fibroblast growth factor; PR, partial response; GEA, gastroesophageal cancer; SD, stable disease; GC, gastric cancer.

Bemarituzumab is a humanized immunoglobulin G1 (IgG1) monoclonal antibody specific to FGFR2b (a splice-variant) that blocks FGF7, FGF10, and FGF22 ligand binding. In the phase I study, bemarituzumab seems to be well tolerated and demonstrates single agent activity as late-line therapy in GC patients. A phase III trial is currently evaluating Bemarituzumab in combination with chemotherapy (FOLFOX6) as front-line therapy for patients with FGFR2b-overexpressing advanced gastroesophageal cancer (FIGHT trial, Five Prime).

As FGFs are rich in gastric cancer tissues, another strategy is the use of FGF traps which can neutralize FGF and reduce cancer cell malignancy. FGF ligand traps are a fusion of an immunoglobulin Fc fragment and a soluble FGFR extracellular domain that competitively binds with FGF1, 2, 3, 7, and 10 to suppress ligand-dependent FGFR signaling. For example, FP-1039 (GSK3052230) is a soluble fusion of the extracellular ligand-binding domain of FGFR1 linked to a modified hinge and native Fc regions of IgG1. FP-1039 was well tolerated, even when used in combination with chemotherapy for lung cancer patients (94). Although there is no clinical data regarding FP-1039 for GC patients, it may be promising considering that FGF7 is possibly a critical player in TME of SGC.

Another FGF trap, the extracellular NSC12, can be used as an FGF antagonist in anti-angiogenic treatment with anti-vascular endothelial growth factor.

### FGFR Inhibitors

According to their target specificities, FGFR kinase inhibitors can be divided to FGFR1/2/3 inhibitors, FGFR4 inhibitors, pan- FGFR inhibitors or multi- kinase FGFR inhibitors. Out of all the FGFR inhibitors, we summarize here the inhibitors which have been tested in clinical trials for gastric cancer patients or some other types of solid tumors.

AZD4547 is a selective FGFR1/2/3 inhibitor which was preclinically tested in FGFR2 amplified SNU16 and SGC083 (GC cell lines) xenograft models, showing positive results. The randomized phase II SHINE study (NCT01457846) investigated whether AZD4547 administered as second-line treatment for advanced GC patients with FGFR2 polysomy or gene amplification improved survival outcome compared to paclitaxel treatment (95). As a result, AZD4547 did not significantly improve progression-free survival (PFS) compared to paclitaxel in these patients. However, the lack of correlation between *FGFR2* amplification/polysomy and FGFR2 expression together with significant intratumor heterogeneity for FGFR2 gene amplification indicate the need for further development of predictive biomarkers.

BGJ398 is another selective FGFR1/2/3 inhibitor, which was identified from integrative analysis of the Cancer Cell Line Encyclopedia (96). Promising results were shown in phase I study for patients with advanced solid tumors, where antitumor activity was demonstrated in patients with *FGFR1*-amplified lung cancer and *FGFR3*-mutant bladder or urothelial cancer (97). However, the study did not include GC patients. *In vitro*, we have shown that BGJ398 significantly decreases the growth of SGC patient-derived OCUM-14 cells (38). Therefore, BGJ398 could be promising for SGC. A small phase I study for patients with advanced solid tumors having alterations of FGFR pathway has been completed (NCT01697605), and the result regarding SGC is awaited from the study.

E7090 is another potent FGFR1/2/3 inhibitor. *In vitro*, E7090 treatment inhibited the phosphorylation of FGFR2 as well as FRS2, ERK1/2, and AKT in SNU-16 GC cells. Moreover, E7090 also had antitumor activity in SNU-16 xenograft mouse model (98). Clinically, the phase I study showed that it has manageable safety profile in patients with advanced solid tumors (99). A phase II study for patients with cholangiocarcinoma is in progress, and the application for gastric cancer may be relevant in the future.

LY2874455 is a reversible pan-FGFR inhibitor that competes for the ATP-binding pocket the kinase domain. A phase I study to determine optimal phase II dose was performed (NCT01212107). Among 29 patients with GC enrolled in the study, one patient was reported to show partial response (PR; more than 30% reduction of the tumor size from the baseline), while 12 patients had best overall response of stable disease (SD; increase in tumor size by at least 20% from the baseline) (100). The median PFS in the GC group was 62.0 days. LY2874455 in combination with other agents should be investigated in the future.

JNI-42756493 (Erdafitinib) is another potent pan-FGFR inhibitor. The first human study reported that Erdafitinib had a manageable safety profile, although it has not been shown whether the study included GC patients (101). Another phase I study was performed in patients with advanced or refractory solid tumors in Japan, and this study includes two GC patients among 19 enrolled patients. This study concludes that Erdafitinib was well tolerated (102). The new phase II study is ongoing to evaluate the efficacy of Erdafitinib in which overall response rate [ORR; the rate of complete response (CR) and PR] is the primary outcome for participants with advanced solid tumors with FGFR mutations and gene fusions (NCT4083976).

Lucitanib is a potent, oral inhibitor of FGFR1, 2, vascular endothelial growth factor receptor types 1, 2, and 3 (VEGFR), platelet-derived growth factor receptor types α and β (PGFRα/β). Lucitanib showed promising efficacy and a manageable profile of adverse events. Clinical benefit was shown in both FGF-aberrant and angiogenesis-sensitive patients (103). A comprehensive phase II program has been planned. Although the reported phase I study did not include GC patients, it might be a promising drug for targeting FGFR2 in a subpopulation of GC.

ARQ087 (Derazantinib) is another multi kinase inhibitor, and it works as pan-FGFR inhibitor. In phase I study, ARQ087 had manageable toxicity at the recommended phase II dose (RP2D) of 300 mg once a day, showed pharmacodynamic effects, and achieved objective responses, particularly in patients with FGFR2 genetic alterations (104). A further study for patients with intrahepatic cholangiocarcinoma is ongoing.

Dovitinib is also a potent multi-kinase inhibitor, and was evaluated in a phase I study of 35 solid tumors including two GCs (105). It was unsatisfactory that neither patient with GC had SD for more than 4 months. On the other hand, three phase II studies of Dovitinib are ongoing in GC (GASDOVI-1, NCT01719549).

Albeit according to preclinical data FGFR alterations were expected to be predictive of responsiveness to FGFR targeted therapies, FGFR alterations alone are not sufficient biomarkers for selecting patients for monotherapies using FGFR-targeted agents. Expression of FGFR mRNA or protein might be more helpful than FGFR amplification for predicting sensitivity to FGFR inhibitors in future studies.

### TGFβ

TGFβ in tumor-stroma interactions favors tumor progression through mechanisms that are still unclear and controversial (106). Before cancer initiation and during the early phase of carcinogenesis, TGFβ can function as a tumor suppressor. Conversely, during the advanced stages, cancer progression and metastasis are promoted by TGFβ signaling (107). To clarify the clinical efficacy of TGFBR inhibitors on the progression of GC at both the early and advanced stages additional studies are needed.

Tranilast [N-(3,4-dimethoxycinnamoyl) anthranilic acid] is a drug which is used clinically for the treatment of excessive proliferation of fibroblasts. By blocking the interactions between fibroblasts and SGC cells, it reduces GC growth and induces cancer cell apoptosis (71). Tranilast not only inhibits fibroblast proliferation but also the release of growth-promoting factors from fibroblasts and cancer cells, and the interactions between these cells (108). In addition, Tranilast and cisplatin combinatorial treatment reduces tumor size, fibrosis, and mitosis, and increases apoptosis in SGC xenograft model (109). Furthermore, the invasion-stimulating ability of fibroblasts is suppressed by Tranilast through inhibiting the production of MMP2 and TGFβ in fibroblasts (110).

Another study also supports the hypothesis that Tranilast could be a new strategy to decrease fibrous tumor represented by peritoneal dissemination. In this study, human peritoneal mesothelial cells (HPMCs) were used to investigate the effects of Tranilast treatment on cells and a xenograft mouse model of fibrosis. TGFβ-mediated EMT-like changes in HPMCs were inhibited in a dose-dependent way by Tranilast treatment through inhibition of Smad2 phosphorylation. In the mouse model, Tranilast significantly decreased tumor size and inhibited fibrosis, compared with the control group proliferation and invasion (111).

Considering these preclinical studies, Tranilast may be a promising novel drug to decrease proliferation and invasion stimulation between fibroblasts and SGC cells (108).

In spite of many preclinical studies about TGFβ, there is still a difficulty in using TGFβ inhibitors clinically. Because TGFβ is a potent inhibitor of epithelial cell proliferation, it could be better to use TGFBR inhibitors with other chemotherapeutic drugs or molecular targeted agents in the future.

### c-Met

Considering the progression mechanism of SGC, c-Met could be a targetable molecule in a subgroup of patients with SGC. Rilotumumab (monoclonal antibody for HGF) showed greater activity than placebo in phase II trial (112). However, it did not improve clinical outcomes in MET positive gastroesophageal cancer in phase III trial (113).

AMG337 is a small molecule MET inhibitor, and it showed promising efficacy in MET-amplified gastroesophageal cancer (114). The result of phase III trial is awaited.

## Future Perspectives

Immune checkpoint inhibitors (ICIs), such as atezolizumab, avelumab, durvalumab, nivolumab, and pembrolizumab, are monoclonal antibodies (mAbs) that block the interaction between programmed cell death protein 1 (PD-1) and its ligand PD-L1, preventing an immunosuppressive signaling cascade. Even though some subsets of patients showed a complete response to these agents (CR rate of 1.1%) (115), the ORR of unselected patients with GC who received anti-PD-1 mAbs remains approximately 10% (116).

Distinct FGFR3 alterations and FGFR3 upregulation were specifically detected in non T cell- inflamed TMEs and associated to resistance against ICIs (117). The specific FGFR inhibitor erdafitinib when administered with anti-PD-1 mAbs in mouse models of FGFR2-driven cancers showed synergistic antitumor outcomes (118), implicating that the combination of FGFR inhibitors and ICIs could be a promising strategy.

M7824 (MSB0011359C) is a novel bifunctional fusion protein formed by an anti-PD-L1 mAb fused with the extracellular domain of TGFβ receptor II, which works as a TGFβ trap (119). In phase I trial, 5 among 31 patients showed PR with manageable safety profile. The combination therapy targeting both TGFβ and ICIs can become a promising treatment strategy for SGC in future.

As described in the previous section, exosomes work as an important messenger between cancer cells and CAF. Therefore, blocking the signaling which starts from exosomes could also be a promising strategy. For example, silencing of exosomal miR-21-5p could block MMT by attenuating TGFβ/SMAD pathway. However, we should confirm which molecules, including miRNAs, are critical players in the TME. Further pre-clinical studies will be needed in developing strategies to target exosomes.

## Conclusion

CAFs communicate with SGC cells in a number of ways, and contribute to the progression of SGC mainly by FGF/FGFR and TGFβ/SMAD signaling axes. In this review we have collected evidence from SGC studies but also from common GC reports and thus we highlight here the need for more basic research in SGC to fully understand the mechanisms of SGC-CAF communication.

Many drugs targeting FGF/FGFR and TGFβ/SMAD signaling pathways have been tested in clinical settings. Among them, bemarituzumab (monoclonal antibody specific to the splice variant FGFR2b) showed 19.0% of ORR in patients with late-line gastroesophageal cancer with FGFR2b expression, and is the only drug being evaluated in phase III clinical trial. Considering the significant role of FGFR2 expression in SGC, a promising treatment strategy will be to use bemarituzumab for SGC cohort. Ultimately, combination therapy targeting multiple players involved in the communication between CAF and SGC cells could improve the survival outcome of patients with SGC in the future.

## Author Contributions

Concept, drafting: YM. Supervision: MY, MO, and KL. Figure making: YM, LM-G, and AS, editing the manuscripts: YM, MY, LM-G, and KL. All authors contributed to the article and approved the submitted version.

## Funding

This work is supported by Swedish Cancer Society CAN 2018/858 (for KL), and Postdoctral research fellowship from the Uehara Memorial Foundation (for YM).

## Conflict of Interest

The authors declare that the research was conducted in the absence of any commercial or financial relationships that could be construed as a potential conflict of interest.
